# Sunflower seeds classification based on sparse convolutional neural networks in multi-objective scene

**DOI:** 10.1038/s41598-022-23869-4

**Published:** 2022-11-18

**Authors:** Xiaowei Jin, Yuhong Zhao, Hao Wu, Tingting Sun

**Affiliations:** 1 School of Information Engineering, Inner Mongolia University of Science and Technology, Baotou, 014010 China; 2 School of Information and Telecommunications Engineering, Dalian Minzu University, Dalian, 116600 China

**Keywords:** Image processing, Classification and taxonomy, Computer science

## Abstract

Generally, sunflower seeds are classified by machine vision-based methods in production, which include using photoelectric sensors to identify light-sensitive signals through traditional algorithms for which the equipment cost is relatively high and using neural network image recognition methods to identify images through cameras for which the computational cost is high. To address these problems, a multi-objective sunflower seed classification method based on sparse convolutional neural networks is proposed. Sunflower seeds were obtained from the video recorded using the YOLOv5 Object detection algorithm, and a ResNet-based classification model was used to classify the seeds according to differences in appearance. The ResNet has the disadvantages of having numerous parameters and high storage requirements; therefore, this study referred to the Lottery Ticket Hypothesis and used the Iterative Magnitude Pruning algorithm to compress the sunflower seed classification model, aiming to ascertain the optimal sparse sub-network from the classification model. Experiments were conducted to compare the effects on model performance before and after pruning, pruning degree, and different pruning methods. The results showed that the performance of the ResNet-based sunflower seed classification model using global pruning was the least affected by pruning, with a 92% reduction in the number of parameters, the best accuracy is 0.56% better than non-pruned and 9.17% better than layer-wise pruning. These findings demonstrate that using the Iterative Magnitude Pruning algorithm can render the sunflower seed classification model lightweight with less performance loss. The reduction in computational resources through model compression reduces the cost of sunflower seed classification, making it more applicable to practical production, and this model can be used as a cost-effective alternative to key sunflower seed classification techniques in practical production.

## Introduction

Sunflower is a cash crop widely grown worldwide and its seeds are divided into oil extraction and edible types. China’s annual sunflower planting area in 2019 was 85.00 million hm^2^, ranked 6th in the world^1^, and the demand for edible sunflower seeds is increasing year by year.

The National Standards of the People’s Republic of China for Sunflower Seeds (GB/T 11764-2008) stipulate the quality requirements and grading standards for sunflower seeds. Sunflower seeds’ classification and grading are important tasks in practical production. Efficient and accurate classification of sunflower seeds can better support processing, packaging, as well as subsequent pricing and marketing, thus promoting the production and marketing of sunflower seeds as well as raising economic benefit.

Sunflower seed classification methods are divided into manual sorting and machine vision-based classification methods. The efficiency and accuracy of manual sorting is lower than machines, and modern industry requires automated systems to reduce costs and improve efficiency. In practical production, a traditional machine vision method is always used to classify sunflower seeds using photoelectric sensors^2,3^. Charged-coupled devices (CCDs) in photoelectric sensors use a photoelectric principle to detect substandard individuals in large piles of bulk material. However, photoelectric sensors are expensive and consume large amounts of electricity, which is not conducive to their application in practical production.

For sunflower seed classification tasks, neural network image recognition methods based on machine vision mostly use machine learning or deep learning algorithms. Commonly, researchers resolve the above problems by machine learning algorithms^4^, such as Support Vector Machines (SVM) algorithms^5,6^ and K-nearest-neighbors (KNN) algorithms^7,8^, etc. JayaBrindha et al.^9^ used ant colony optimization techniques to optimize the order of cascaded SVM by maximizing the total probability of correct decisions for the sunflower seed classification. A region-oriented seed-based segmentation (ROSS) method was proposed by Bantan et al.^10^ to enhance the dataset and retain the maximum amount of information in each sunflower seed image in order to select the non-overlapping regions to be analyzed. Multispectral features of the region to be analyzed were extracted by a multispectral radiometer (MSR5), which was fused with texture features. The fused and optimized multi-feature dataset was deployed on four supervised classifiers for seed recognition. Çetin et al.^11^. classified and evaluated the performance of six sunflower seed varieties by six different machine learning algorithms, with RF, SVM and MLP having the highest best accuracy values of 80.16, 79.68 and 78.89 respectively. However, as the above classification methods are based on machine learning algorithms, most of them for image classification require multiple steps, i.e., feeding the features into a classifier for classification by feature extraction and feature selection, which is a tedious step, whereas deep learning can integrate the above steps^12–14^.

Kurtulmuş^15^ used deep learning methods to classify sunflower seeds for the first time, identifying four types of sunflower seeds by three popular deep learning architectures: AlexNet^16^, GoogleNet^17^ and ResNet^18^, whereas GoogleNet achieved the highest accuracy of 96% in classification. Luan et al.^19^ used a CNN model with eight convolutional layers to extract image features and added an adaptive channel attention mechanism to recalibrate channel-based features by considering the dependencies between channels in order to enhance image features that are crucial for the classification task in order to increase the accuracy of sunflower seed classification. However, most of the deep learning models used for classification suffer from a large number of parameters, high computational cost, and high storage requirements, which are not conducive to applying in practical production.

Researchers are constantly looking into different methods to improve the accuracy of models. To address instability and errors in packet classification tasks, Hartpence et al.^20^ solved complex communication networks by integrating multiple models and using voting strategies and redundant decisions. After training and tuning, the model could achieve 99% accuracy in the general and UDP phases, achieved 94% accuracy in the TCP phase, not only reducing training time but also improving the accuracy of the model. Gu et al.^21^ propose a novel link prediction-based network representation that not only learns meaningful node representations but also achieves high accuracy in node centrality measurement, community detection, and link prediction tasks, and also demonstrates its effectiveness in real-world networks through experiments. Through supervised learning, Zhao et al.^22^ forecast past data summaries directly from the data distribution and utilize the new data to cluster the past summaries. The results of experiments demonstrate that this method surpasses previous incremental face clustering techniques, increasing incremental face clustering accuracy while decreasing processing time. Palmer et al.^23^ compared several multi-label/multi-objective methods with single-label methods in order to classify wines according to price, grade quality, and provenance in a multi-label manner. The experimental results show that the Bayesian classifier chain produced better overall results.

Frankle et al. proposed the Lottery Ticket Hypothesis (LTH) for finding optimal sub-networks by the Iterative Magnitude Pruning (IMP) algorithm^24^. This approach substantially reduces the number of parameters required in the inference process without affecting the performance of the model, reducing the storage requirements and computational costs, thus allowing significant cost savings and making the model more suitable for application in practical production. In the Lottery Ticket Hypothesis, a sparse sub-network with the following properties is found in the randomly initialized feedforward neural network. When trained independently, this sparse sub-network is able to achieve an accuracy similar to the original network after at most the same number of iterations as the original network.

In order to reduce the number of parameters of the classification model, reduce the computational cost and achieve the classification of sunflower seeds using sparse networks in multi-object scenes, a sunflower seed classification method based on sparse convolutional neural networks is proposed in this paper. In the experiments, the YOLOv5 object detection algorithm was used to obtain sunflower seed images, and the images were used to construct a sunflower seed classification dataset. Then constructed convolutional neural networks (CNN), and classification models were trained using the sunflower seed classification dataset by CNN. According to the LTH, the sunflower seed classification model was pruned by the IMP algorithm to pick out a sparse sub-network that is close to the performance of the original model, so as to achieve the compression of the sunflower seed classification model to reduce the cost of sunflower seeds in classification processing. The effects of before and after pruning, pruning degree, and different pruning methods on the performance of the sunflower seed classification model were also investigated, so as to obtain the sparse sub-network with the least effect on the performance of the classification model under the condition of substantially pruned parameters.

## Materials and methods

### Sunflower seeds

The variety of sunflower seeds used in the experiment is CF363, which is grown on a farm on the outskirts of *Chifeng, Inner Mongolia, China*. And we got the sunflower seed product from a seed processing factory in September 2020. 500 kg of sunflower seeds were randomly selected from there.

### Video recorded acquisition

**Figure 1:**
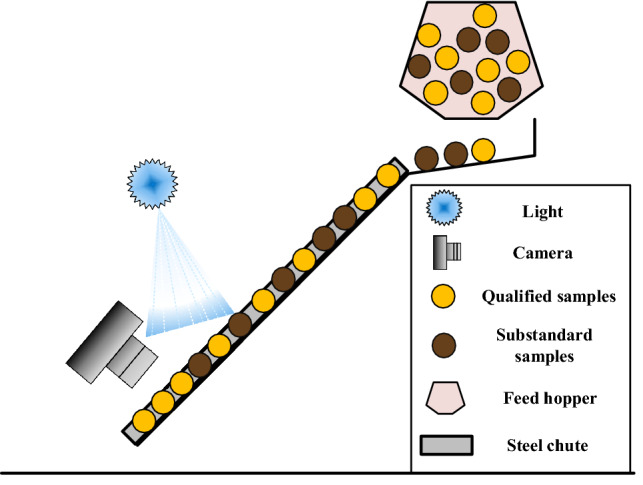
Sunflower seed classification scene simulation chamber.

To simulate the sunflower seed classification scene in practical production, a classification scene simulation chamber was constructed with artificial lighting conditions. An inclined ramp with an angle of 45° was built under the light in the simulation chamber, and a steel chute with a length of 1 m, a width of 30 cm, and a height of 10 cm was fixed to the ramp. A feed hopper was placed at the top of the steel chute at a distance of 20 cm from the chute to ensure that the sunflower seeds were fed into the chute in a uniform and continuous manner. A camera was placed 20 cm above the chute as a video recorded acquisition device. The 500 kg of sunflower seeds were mixed well, and 5 kg of them were acquired at a time and poured into the feed hopper at constant speed while the video recording was started. After the end of each video recording, 5 kg of sunflower seeds were obtained again for the next shot. A total of 100 recorded videos were captured, each lasting 5 min. The frame width, frame height, and frame rate are 1920p, 1080p, and 240.37f/s. The simulation chamber is shown in Fig. 1.

### Research plan

**Figure 2:**
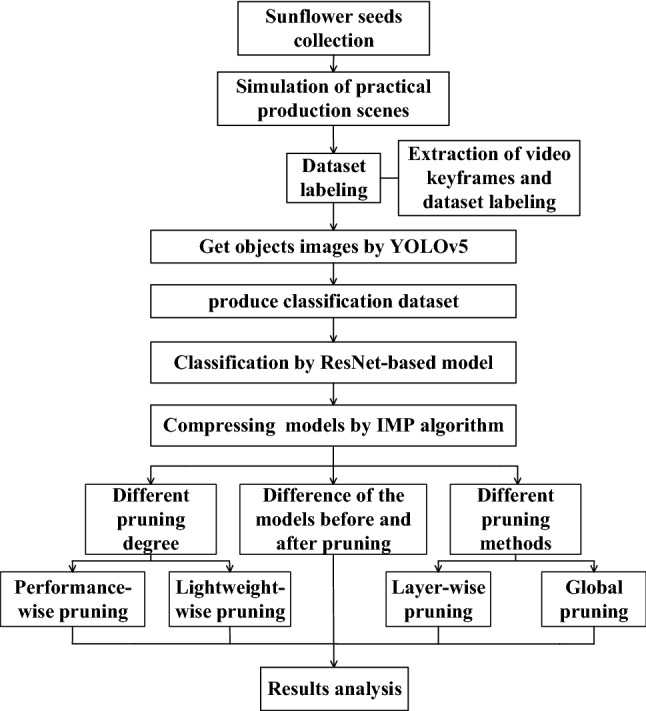
The research plan for this study.

TO achieve a cost-effective alternative to traditional sunflower seed classification methods in practical production, this study trained a sparse sub-network with a performance close to the original model. A CNN was first constructed and trained to complete the classification task on the sunflower seed classification dataset, and then the sunflower seed classification model was compressed by the IMP algorithm. By comparing the effects of before and after pruning, pruning degree, and different pruning methods on the performance of the model, it was demonstrated that using the global pruning method to compress the ResNet-based sunflower seed classification model could achieve the maximum retention of the performance of the original network while reducing the complexity of the network. The research plan is shown in Fig. 2 and consists of five components.

#### Constructing the dataset

Sunflower seed object images were detected from the video recorded by the YOLOv5 object detection algorithm, and used to build the sunflower seed classification dataset.

#### Sunflower seed classification

A ResNet-based classification model was used to classify sunflower seeds according to differences in the appearance of sunflower seeds.

#### Compression of the classification model

Compress sunflower seed classification models by the IMP algorithm.

#### Exploring the impact of the pruning degree on model performance

The sunflower seed classification model was subjected to ‘Performance-wise pruning’ and ‘Lightweight-wise pruning’, respectively. The effects of different pruning degrees on classification performance were compared.

#### Analysis of the effect of different pruning methods on model performance

The ResNet-based sunflower seed classification model was compressed by layer-wise pruning and global pruning, respectively. The effects of different pruning methods on the classification performance were compared.

### Acquisition of sunflower seeds object images in multi-object scene

In order to meet the data sample needs of the sunflower seed classification model, the sunflower seed objects should be detected first. As sunflower seeds are small targets, an object detection algorithm suitable for detecting small objects is necessary to obtain sunflower seed object images.

**Figure 3:**
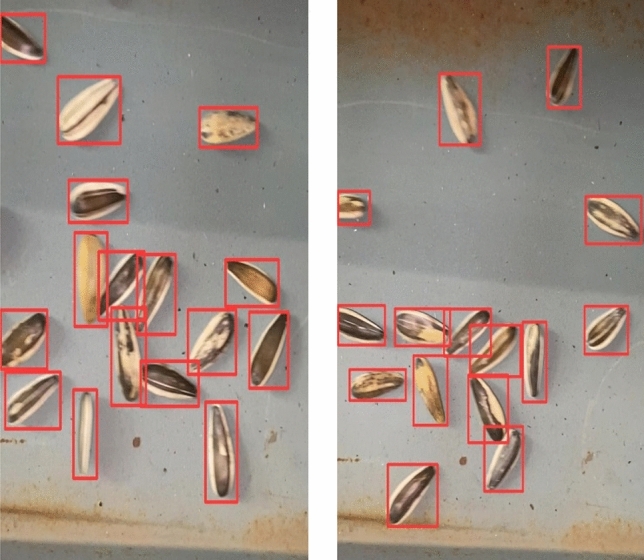
Object detection video capture.

10 videos were randomly selected for object detection from the 100 videos taken, and the object detection video capture is shown in Fig. 3.

**Figure 4:**
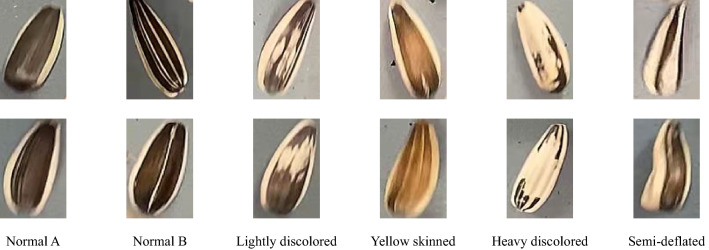
Sunflower seed samples.

A batch of object images were extracted per 10 frames. Based on the appearance and color of sunflower seeds, the acquired sunflower seed object images were classified into 6 categories: Normal A, Normal B, Lightly discolored seeds, Yellow skinned seeds, Heavy discolored seeds, and Semi-deflated seeds. To ensure a balanced sample, 1000 images were randomly selected for each category, making a total of 6000 images, from which 600 images were randomly selected as the validation set, 600 images as the test set, and the remaining 4800 images as the training set. Two randomly selected sunflower seed object images from each class were used as sample images. The sample images of sunflower seeds are shown in Fig. 4.

### Sunflower seeds’ object detection method

The YOLOv5 was used to obtain sunflower seed object samples. YOLOv5 is based on the original YOLO model^25–27^, with optimization of data pre-processing, feature extraction, feature fusion, backbone network, and loss function. The model has the advantages of small volume and fast training compared to other object detection methods. The Mosaic data enhancement method^28^ is used in the data pre-processing stage. The Mosaic method stitches four images into the training data after random cropping, an improvement in its ability to detect small objects.

Since deeper feature maps carry more semantic information and less positional information, and the opposite for shallow feature maps. Therefore, it is not sufficient to use only the Feature Pyramid Network (FPN)^29^, which conveys semantic information from the top down. YOLOv5 adds the Path Aggregation Network (PAN)^30^ after the FPN layer, which conveys positional information from the bottom up. Above improvement allows for further integration of bottom and top levels information and handles the multi-scale variations problem in object detection with a small increase in computational effort.

**Figure 5:**
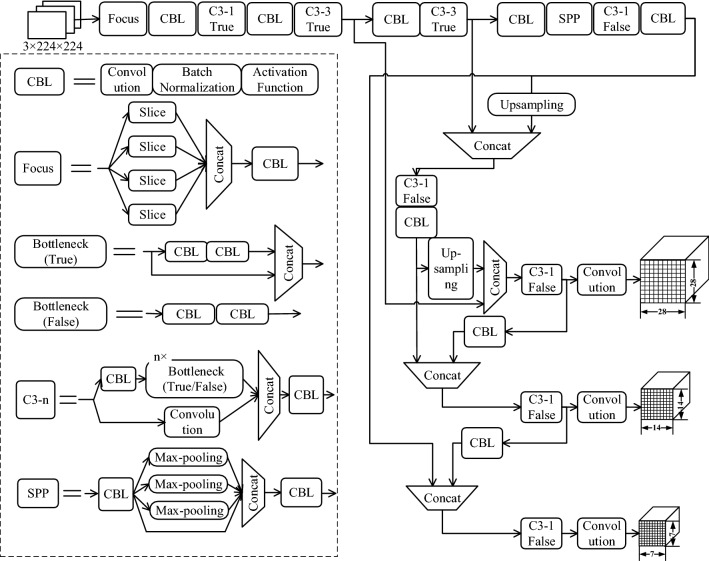
Object detection model for sunflower seeds based on YOLOv5.

Figure 5 shows the structure of the sunflower seed object detection model based on YOLOv5. The “CBL” module is composed of Convolution + Batch normalization + Leaky Relu, and the extended structure of this module is illustrated in the first image inside the dashed box in the lower left corner of Fig. 5. The “Focus” module represents the Focus layer proposed in YOLOv5. It is a special down-sampling technique that uses a slicing operation to split the high-resolution feature map into numerous low-resolution feature maps and then performs a convolution operation after stitching together the multiple feature maps. This method can reduce the information loss caused by down-sampling. The second image in the dashed box in the lower left corner of Fig. 5 illustrates the method’s unfolding structure. The “C3-n True/False” module in Fig. 5, which is based on the CSPNet structure, consists of a CBL module, n Bottleneck modules, and a convolutional layer. The fifth image in the dashed box in the lower left corner of Fig. 5 illustrates the method's unfolding structure. In the C3-n structural diagram, the “Bottleneck True/False” module successfully integrates Bottleneck and normal convolution by combining “True/False”, resulting in less code and a clearer design. The third and fourth images in the dashed box at the bottom left of Fig. 5 depict the extended structure. The ‘SPP’ module in Fig. 5 is Spatial pyramid pooling, which converts a feature map of any size into a fixed-size feature vector. The expanded structure is shown in the 6th image of the dashed box in the bottom left corner of Fig. 5.

### Sunflower seeds classification

In order to ensure that the model retains the same maximum performance as the original model even after compression, we proposed a method for classifying sunflower seeds by a ResNet-based classification model. It could reduce the impact of pruning on model performance using the short-circuiting mechanism of ResNet^31^. The core idea of ResNet is to increase the residual units by the short-circuiting mechanism to achieve long-range transmission of information. It can solve the degradation problem of the network and allow neural networks to adopt deeper designs. ResNet has a recursive nature and integration properties. The recursive nature is shown in Eq. (1), where the output of each residual block is based on the combination of two sub-blocks.

1 $${y}_{i}\equiv {f}_{i}\left({y}_{i-1}\right)+{y}_{i-1},$$

where ${y}_{i}$ is the output of layer i, ${f}_{i}$ is the convolution sequence for layer i, and ${y}_{i-1}$ is the output of layer i−1 (as input to layer i).

To better express the integration properties of the ResNet, a ResNet with three residual blocks from input y_0_ to output y_3_ is used as an example, and Eq. (2) is a recursive expression of the ResNet with those three residual blocks. Equation (2) is expanded to Eq. (4) to make the integration structure of the ResNet more apparent.

2 $${y}_{3}={y}_{2}+{f}_{3}\left({y}_{2}\right),$$

3 $${y}_{3}=\left[{y}_{1}+{f}_{2}\left({y}_{1}\right)\right]+{f}_{3}\left({y}_{1}+{f}_{2}\left({y}_{1}\right)\right),$$

4 $${y}_{3}=\left[{y}_{0}+{f}_{1}\left({y}_{0}\right)+{f}_{2}\left({y}_{0}+{f}_{1}\left({y}_{0}\right)\right)\right]+{f}_{3}\left({y}_{0}+{f}_{1}\left({y}_{0}\right)+{f}_{2}\left({y}_{0}+{f}_{1}\left({y}_{0}\right)\right)\right),$$

where ${y}_{0}$ is model inputs, ${y}_{n}$ is the output of layer n, and ${f}_{n}$ is the convolution sequence for layer n.

**Figure 6:**
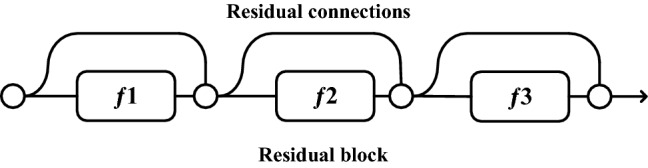
3-Block ResNet.

**Figure 7:**
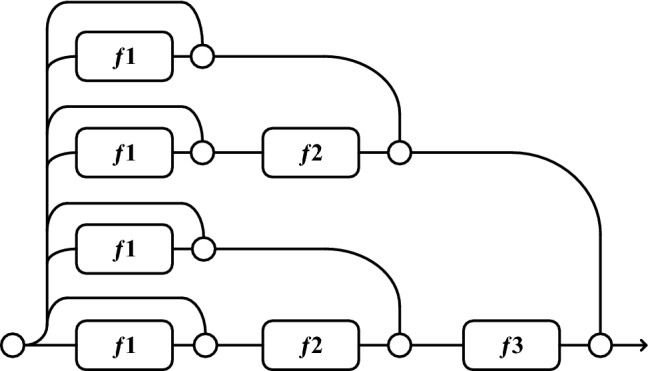
Unraveled view for 3-Block ResNet.

ResNet has O^(2n)^ implicit paths connecting inputs and outputs. Adding a residual block will double the number of implicit paths, as shown in Figs. 6 and 7. Figure 6 shows a ResNet with three residual blocks built according to Eq. (2). Figure 7 shows an expanded view of Fig. 6 according to Eq. (4), where the circular nodes indicate addition.

**Figure 8:**
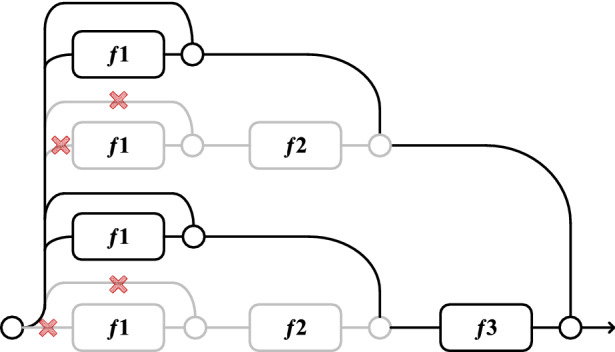
Deleting some paths in unraveled view for 3-Block ResNet.

In traditional CNNs, the input always flows in a single path from the first layer to the last, removing the network structure changes the unique path from input to output, deactivating the neurons on those paths and changing the distribution of all subsequent layers, resulting in a reduced model performance. However, a ResNet network is an integrated model assembled from a collection of paths. There is low dependence among these paths and the performance of the residual network is not significantly affected when deleting some layers from the ResNet network (i.e., discarding some of the paths), as shown in Fig. 8.

In order to verify the effectiveness of the classification and compression methods in this paper, some classical CNNs were also used as comparisons, as shown in Table 1.

**Table 1:** Other networks used in experiment as comparisons.

Model	Characteristic
LeNet^32^	LeNet is one of the first convolutional neural networks and is the starting point for a large number of neural network architectures. Its application of convolution to maintain the local correlation of an image and invariance of image translation, scaling and deformation through local receptive fields, shared weights, pooling, etc. As an end-to-end model, LeNet does not require the use of multiple steps for classification work as in traditional machine learning
AlexNet	AlexNet applied Rectified Linear Unit (ReLU)^33^ for the first time in CNN to solve the gradient disappearance problem that often occurs when using Sigmoid and to improve the computational speed, as well as random discarding and data augmentation to solve the overfitting problem
DenseNet^34^	Instead of widening the network structure and deepening the number of layers to improve the network performance, DenseNet innovatively uses feature reuse and bypass to make the model highly parametric efficient. This approach not only reduces the number of parameters and effectively suppresses overfitting but also alleviates the problem of gradient disappearance to a certain extent

### IMP algorithm based on LTH

The redundant parameters, which have no positive impact on the final output, have been one of the main drawbacks of deep learning models for a long time. A neural network compression technique that reduces model parameters and improves inference performance by removing these redundant parameters is called pruning.

LTH proposes that by using the IMP algorithm on a randomly initialized feedforward network, a re-trainable sparse sub-network could be found. This sub-network has only 5–10% of the original parameters left after multiple pruning, but similar performance as that. Such a sparse sub-network is called a “Winning Ticket”. In addition, networks that have been moderately pruned (minus 50–80% of the number of parameters) tend to outperform the non-pruned model.

The method in this study pruned unnecessary connections that have little impact on the performance of the network, i.e., weights were evaluated to be of minimal rank. The IMP determined which parameters could be pruned out without affecting the performance of the model. More and more connections were pruned, the remaining connections forming the “Winning Ticket” architecture. Applying the IMP algorithm, only a small number of weights were pruned after each pruned iteration and then evaluated and pruned periodically to reduce the impact of noise on the overall model. The network could only be trained well if the initialization weights of the original network were used when initializing the sub-networks; re-initializing the weights would result in poor model training. During the training process, the pruning process was implemented by a binary mask that set all weights smaller than a preset threshold to 0 and frozen them so that the corresponding connections were no longer involved in the training.

**Figure 9:**
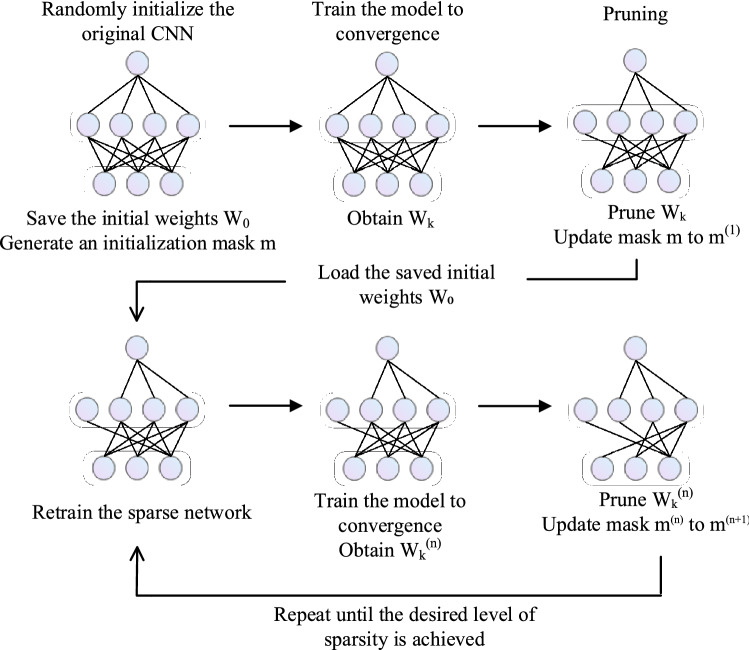
IMP schematic.

The steps used in this study to filter the optimal sparse sub-network from the CNN-based sunflower seed classification model that is close to the performance of the original model using the IMP algorithm are as follows:

1. Randomly initialize the original CNN and save the initial weights W_0_.
2. Generate an initialization mask m.
3. Train the model to convergence using the sunflower seed dataset to obtain the model’s weights W_k_.
4. Prune the parameters in W_k_ according to the pruning rate (pruning rate is a hyperparameter) and update the mask m.
5. Initialize the network by the initial weights saved in step (1) and retrain the sparse network.
6. Repeat steps (3)–(5) until the desired level of sparsity is achieved, or the accuracy of the model is significantly reduced. The above process is shown in Fig. 9.

## Results and discussion

The experimental environment in this study was a 64-bit Ubuntu system, an Intel Xeon Gold 6139 processor, and an NVIDIA GeForce RTX 2080Ti GPU. The programming language was Python 3.7, all models were based on Pytorch 1.2.0, and the development tool was PyCharm 2020.

To compare the differences among sunflower seed classification models with different networks, the hyperparameters in Table 2 were kept constant for experiments.

**Table 2:** Hyperparameter configuration.

Hyperparameter	Value
Learning_rate	1.2e−3
Batch_size	60
Print_frequence	10
Prune_percent	10
Prune_iteration	25
End_epoch	150

The experiments were designed to train the models to convergence using the hyperparameters mentioned in the table above. Different training cycles were used to achieve convergent training for LeNet5 and AlexNet as they did not reach convergence within a given 150 training cycles (epoch). The modified hyperparameters are shown in Table 3.

**Table 3:** Modified hyperparameter.

Model	End_epoch
LeNet5	300
AlexNet	250
ResNet18	150
DenseNet121	150

### Sunflower seeds classification result

The performance of sunflower seed classification models based on 4 CNNs were tested by experiment, and a comparison of the best accuracy and the number of parameters of each model was shown in Table 4.

**Table 4:** Comparison of the best accuracy and the number of parameters of each model without pruning.

Model	Best accuracy (%)	The number of parameters (M)
LeNet5	83.61	0.062
AlexNet	83.89	57.01
ResNet18	86.94	11.17
DenseNet121	89.72	6.96

### Model sparsification

As can be seen from Table 4, models used in this study were characterized by a large number of parameters, leading to high computational costs, so the sunflower seed classification model was compressed using the IMP algorithm based on LTH. The experiments set the iterative pruning rate to 10% and pruned after each iteration, with a total of 25 pruned iterations set and each pruned iteration containing 150 epochs (among them, each pruned iteration of LeNet5 contains 300 epochs and each pruned iteration of AlexNet contains 250 epochs).

Analyzing the model structure, the ResNet has integration properties, the dependency between paths is not strong, dropping some paths has little impact on the ResNet, so the ResNet suffers less from pruning.

#### Performance-wise pruning

**Figure 10:**
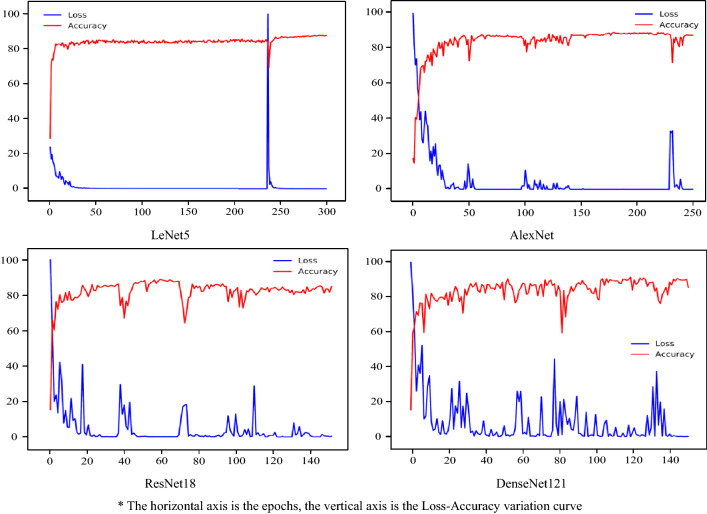
Loss—accuracy value for models with performance-wise pruning.

Since pruning can impact model performance, this section conducts Performance-wise pruning experiments on the models with maximum assurance of model performance. Figure 10 shows the Loss-Accuracy curves of each model when the best accuracy emerges during the pruned processing, where LeNet5 is chosen after the 10th pruned iteration; AlexNet is chosen after the 16th pruned iteration; ResNet18 is chosen after the 12th pruned iteration; and DenseNet121 is chosen after the 8th pruned iteration.

According to Fig. 10 and Table 5, the models with performance-wise pruning (remaining 20–50% of parameters) outperformed the non-pruned models. The best accuracy of LeNet5, AlexNet, ResNet18, and DenseNet121 all increased by 4.17%, 5.28%, 1.95%, and 1.66%, respectively. According to the experiments, the use of performance-wise pruning with very little constraint on computational resources and cost can both compress the model appropriately and improve its performance.

**Table 5:** Comparative table of various data with performance-wise pruning.

Model	Pruned iterations	Remaining parameters as a percentage (%)	Best accuracy (%)	Best accuracy with non-pruned (%)
LeNet5	10	39.0	87.78	83.61
AlexNet	16	20.6	89.17	83.89
ResNet18	12	31.4	88.89	86.94
DenseNet121	8	48.1	91.38	89.72

#### Lightweight-wise pruning

**Figure 11:**
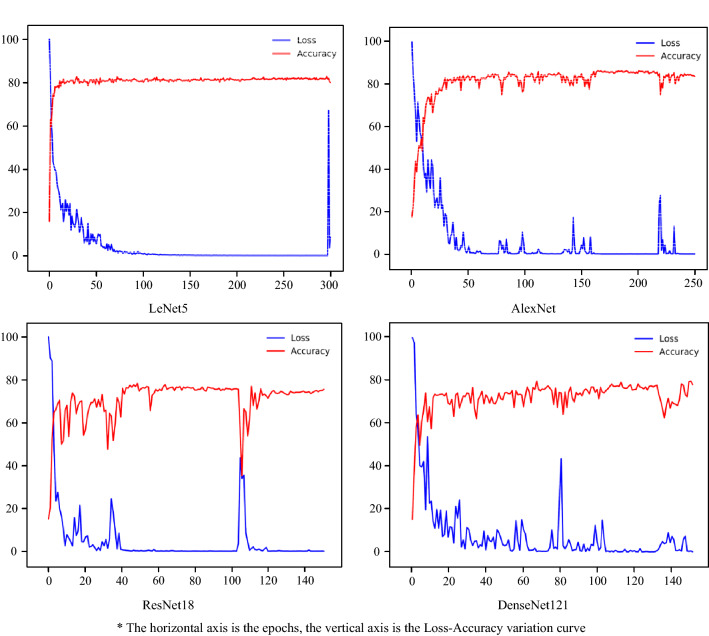
Loss—accuracy value for models with lightweight-wise pruning.

In this section, the Loss-Accuracy curves of each model after the 25th pruned iteration were selected. As shown in Fig. 11.

According to Fig. 11 and Table 6, LeNet5, AlexNet, and DenseNet121, after 25 pruned iterations of pruning, the number of parameters was only 8–10% of the non-pruned model, but the performance was still close to the non-pruned model. Therefore, using lightweight-wise pruning is an effective method when computational resources and costs are very limited.

**Table 6:** Comparative table of various data with lightweight-wise pruning.

Model	Remaining parameters as a percentage (%)	Best accuracy after the 25th pruned iteration (%)	Best accuracy with non-pruned (%)
LeNet5	8.3	82.78	83.61
AlexNet	8.0	86.39	83.89
ResNet18	8.0	78.33	86.94
DenseNet121	8.1	80.01	89.72

In summary, the model after performance-wise pruning was able to achieve the best accuracy of 91.38% with 48.1% of the remaining parameters, and the model after lightweight-wise pruning was able to achieve the best accuracy of 86.39% with 8.0% of the remaining parameters. In practical production, the appropriate pruning level can be chosen by the actual situation of computational resources and costs, so as to meet the needs of the sunflower seed classification task while reducing computational costs and saving computational resources.

### Different pruning methods

It was found that after 25 pruned iterations, the best accuracy of ResNet18 was 78.33%, which was 8.61% lower than the non-pruned one, and did not achieve the expected result.

After analyzing the experimental results, this study concluded that ResNet18 did not achieve the expected accuracy because of the fewer layers of the model, so ResNet50 and ResNet101 were chosen as the comparison experiments in this study, using the hyperparameters in Table 2. After 25 pruned iterations, the comparison table of the best accuracy and the percentage of remaining parameters for ResNet18, ResNet50, and ResNet101 were shown in Table 7, and also compared with the non-pruned model.

**Table 7:** Comparative table of various data after increasing the number of layers.

Model	Remaining parameters as a percentage (%)	Best accuracy after the 25th pruned iteration (%)	Best accuracy with non-pruned (%)
ResNet18	8.0	78.33	86.94
ResNet50	8.1	77.22	85.56
ResNet101	8.1	75.83	85.56

From Table 7, we can see that the performance of ResNets after deepening the number of layers were still not as accurate as expected, so the cause of the performance degradation is not related to the number of layers.

Analyzing the above results, since the pruning method used in this study was layer-wise pruning. The layer-wise pruning prunes a certain percentage of parameters from each layer of the network. For a deep network like ResNet, some layers have far more parameters than others, and when all layers are pruned at the same pruned rate, more important parameters will be pruned in layers that have fewer parameters. Moreover, ResNet increases the residual units by short-circuiting mechanism to achieve long-range transmission of information. And if layer-wise pruning is used, too many parameters will be pruned in the initial layers and in the layers with fewer parameters. After several pruned iterations, only a small number of parameters remain in the initial layers and layers with few parameters, which degrades the performance of the residual structure. So the pruning method used in this study is not suitable for ResNet, therefore the performance of the model degrades after several pruned iterations.

To validate the above analysis, global pruning was used to prune the ResNet for experiments. Global pruning could prune the whole network at the default pruning rate to prevent breaking the residual structure of ResNet. LeNet5, AlexNet, and DenseNet121 were also used as comparisons to demonstrate that ResNet was least affected by pruning.

**Figure 12:**
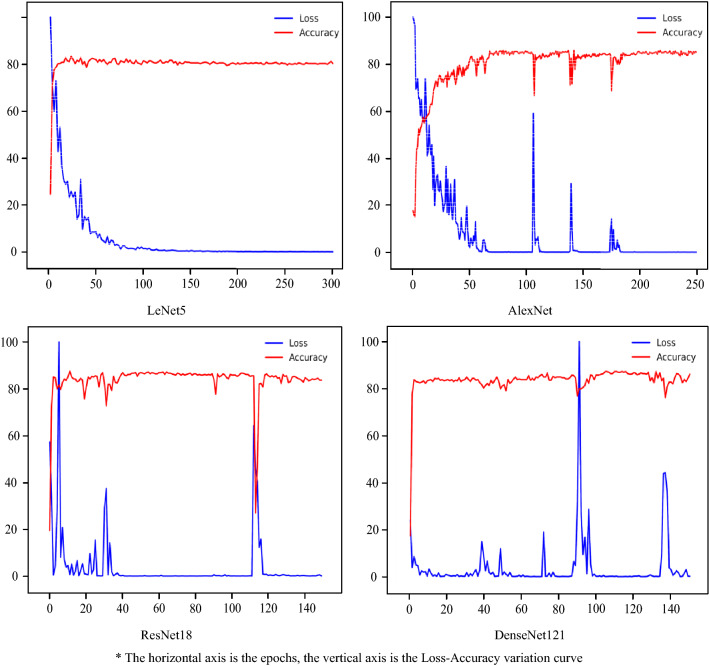
Loss—accuracy value for models with global pruning.

As shown in Fig. 12 and Table 8, after 25 pruned iterations using global pruning, the accuracy of ResNet101 increased by 11.39% compared to using layer-wise pruning, the accuracy of ResNet50 increased by 9.72% and the accuracy of ResNet18 increased by 9.17%. With 8–10% of the parameters remaining, the model performance was still close to the model when non-pruned.

**Table 8:** Comparative table of various data with global pruning.

Model	Remaining parameters as a percentage (%)	Best accuracy with Global pruning (%)	Best accuracy with Layer-wise pruning (%)	Best accuracy with non-pruned (%)
ResNet18	8.0	87.50	78.33	86.94
ResNet50	8.1	86.94	77.22	85.56
ResNet101	8.1	87.22	75.83	85.56
LeNet5	8.3	83.33	82.78	83.61
AlexNet	8.0	84.72	86.39	83.89
DenseNet121	8.6	86.39	80.01	89.72

Table 9 shows a schematic table of ResNet18’s partial layer’s parameters using layer-wise pruning and global pruning, respectively, at a pruning rate of 10%. We can tell from Table 9 that the layers with fewer parameters were over-pruned with layer-wise pruning. In contrast, when using global pruning, the parameters of the less parametric layers were retained due to the overall pruning throughout the network.

**Table 9:** The number of remaining parameters in partial layers with different pruning methods.

Weight name	Total parameters	Layer-wise pruning			Global pruning		
		Pruned parameters	Remaining parameters	Remaining percentage (%)	Pruned parameters	Remaining parameters	Remaining percentage (%)
layer2.0.conv1.weight	73,728	7373	66,355	90	615	73,113	99.17
layer2.0.conv2.weight	147,456	14,746	132,710	90	1295	146,161	99.12
layer2.0.shourcut.0.weight	8192	820	7372	89.99	36	8156	99.56
layer4.0.conv1.weight	1,179,648	117,965	1,061,683	90	69,006	1,110,642	94.15
layer4.0.conv2.weight	2,359,296	235,930	2,123,366	90	247,797	2,111,499	89.5
layer4.0.shourcut.0.weight	131,072	13,108	117,964	90	1483	129,589	98.87

**Figure 13:**
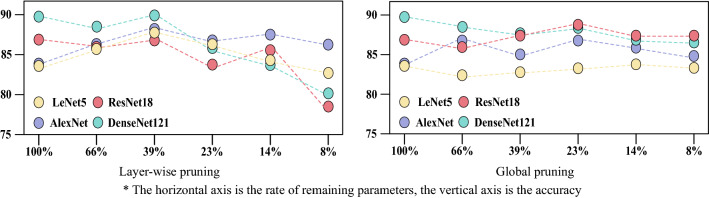
Accuracy and the rate of remaining parameters for each model with different pruning methods.

Figure 13 illustrates a comparison of the accuracy and the rate of remaining parameters for each model when using different pruning methods. According to Fig. 13, LeNet and AlexNet have fewer layers and the number of parameters in each layer has little variation, so the difference in performance of the models using different pruning methods is small. ResNet and DenseNet not only have more layers but also have a greater variation in the number of individual layer parameters, so using layer-wise pruning would disrupt the layers with fewer parameters. In addition, since ResNet contains residual structure, global pruning can better preserve the residual structure, so ResNet is more suitable for global pruning.

## Conclusion

In this work, we simulated the practical production and took CF363 sunflower seeds collected in *Chifeng, Inner Mongolia, China* as data samples. By constructing the simulation chamber, the YOLOv5 object detection algorithm was used to detect sunflower seeds object images from the captured video recorded to construct a sunflower seed classification dataset. A ResNet-based classification model was used to classify sunflower seeds based on differences in the appearance of sunflower seed objects. Based on the LTH, the ResNet-based classification model was compressed by the IMP algorithm to pick out sparse sub-network with similar performance to the original network.

After several experiments, the model applied performance-wise pruning (20–50% of parameters remaining) outperformed the non-pruned model; the model applied lightweight-wise pruning had only 8–10% of parameters left, and the performance was still similar to the non-pruned model, proving that the IMP algorithm based on LTH can compress the model while achieving performance similar to the original one. In practical production, the appropriate level of pruning can be selected according to the actual situation of computational resources and costs, thus reducing the computational resources and lowering the costs required for classification, making it more suitable for practical production under different conditions and further optimizing the sunflower seed grading, pricing, and marketing system.

It was demonstrated that the ResNet-based sunflower seed classification model using global pruning preserved the original performance of the model better than using layer-wise pruning. After using global pruning for 25 pruned iterations, the best accuracy of the sunflower seed classification model was 87.50%, a 9.17% improvement over the compression method using layer-wise pruning. The comparison experiments with other models also demonstrated that the ResNet-based sunflower seed classification model by global pruning had minimal impact on the model performance while reducing the cost of sunflower seed classification, and the selected sparse sub-networks are more robust.

Limited by the speed of the sliding of sunflower seeds in the steel chute, it was difficult to observe some smaller features such as mold spots and worm-eaten holes in the sunflower seed object images. Therefore, the next work will focus on how to obtain non-obvious features in the moving state of sunflower seeds for easily classifying sunflower seeds more finely.

## Data Availability

The data presented in this study are available on request from the corresponding author.
